# Monitoring β-arrestin recruitment via β-lactamase enzyme fragment complementation: purification of peptide E as a low-affinity ligand for mammalian bombesin receptors

**DOI:** 10.1371/journal.pone.0127445

**Published:** 2015-06-01

**Authors:** Yuichi Ikeda, Hidetoshi Kumagai, Hiroaki Okazaki, Mitsuhiro Fujishiro, Yoshihiro Motozawa, Seitaro Nomura, Norifumi Takeda, Haruhiro Toko, Eiki Takimoto, Hiroshi Akazawa, Hiroyuki Morita, Jun-ichi Suzuki, Tsutomu Yamazaki, Issei Komuro, Masashi Yanagisawa

**Affiliations:** 1 Department of Cardiovascular Medicine, University of Tokyo, Tokyo, Japan; 2 Department of Molecular Genetics, Howard Hughes Medical Institute, University of Texas Southwestern Medical Center, Dallas, Texas, United States of America; 3 Department of Diabetes and Metabolic Diseases, University of Tokyo, Tokyo, Japan; 4 Department of Gastroenterology, University of Tokyo, Tokyo, Japan; 5 Department of Advanced Clinical Science and Therapeutics, University of Tokyo, Tokyo, Japan; 6 Clinical Research Support Center, University of Tokyo, Tokyo, Japan; 7 International Institute for Integrative Sleep Medicine (WPI-IIIS), University of Tsukuba, Tsukuba, Japan; Loyola University Chicago, Stritch School of Medicine, UNITED STATES

## Abstract

Identification of cognate ligands for G protein-coupled receptors (GPCRs) provides a starting point for understanding novel regulatory mechanisms. Although GPCR ligands have typically been evaluated through the activation of heterotrimeric G proteins, recent studies have shown that GPCRs signal not only through G proteins but also through β-arrestins. As such, monitoring β-arrestin signaling instead of G protein signaling will increase the likelihood of identifying currently unknown ligands, including β-arrestin-biased agonists. Here, we developed a cell-based assay for monitoring ligand-dependent GPCR-β-arrestin interaction via β-lactamase enzyme fragment complementation. *Inter alia*, β-lactamase is a superior reporter enzyme because of its cell-permeable fluorescent substrate. This substrate makes the assay non-destructive and compatible with fluorescence-activated cell sorting (FACS). In a reporter cell, complementary fragments of β-lactamase (α and ω) were fused to β-arrestin 2 and GPCR, respectively. Ligand stimulation initiated the interaction of these chimeric proteins (β-arrestin-α and GPCR-ω), and this inducible interaction was measured through reconstituted β-lactamase activity. Utilizing this system, we screened various mammalian tissue extracts for agonistic activities on human bombesin receptor subtype 3 (hBRS3). We purified peptide E as a low-affinity ligand for hBRS3, which was also found to be an agonist for the other two mammalian bombesin receptors such as gastrin-releasing peptide receptor (GRPR) and neuromedin B receptor (NMBR). Successful purification of peptide E has validated the robustness of this assay. We conclude that our newly developed system will facilitate the discovery of GPCR ligands.

## Introduction

G protein-coupled receptors (GPCRs), cell-surface receptors containing the characteristic seven-transmembrane domain, respond to various physiological stimuli such as photons, lipids, amines and peptides [1]. Since they play critical roles in many physiological systems, GPCRs serve as target molecules for nearly one-half of clinically approved drugs. Completion of the human genome project led to the discovery of more than 800 GPCRs, and much effort has been made to identify their cognate endogenous and/or synthetic ligands.

Ligand binding triggers a rearrangement of the seven transmembrane alpha helices, and the conformational change of the receptor activates downstream signaling [2]. In the classical signal transduction model, GPCR signaling is limited to the activation of heterotrimeric G proteins. This canonical pathway is terminated by ß-arrestins, which translocate from the cytosol to the activated receptor. Although ß-arrestins were first recognized as a desensitizer of G protein signaling, recent studies have shown that ß-arrestins also function as a multiprotein scaffold that mediates non-canonical GPCR signaling [3]. Thus, GPCRs are currently considered to signal not only through G proteins but also through ß-arrestins.

GPCRs are capable of adopting distinct active conformations depending on the ligands interacting with the receptor [4]. In agreement with this, some ligands selectively activate either a G protein- or ß-arrestin-mediated pathway, demonstrating that these pathways are mutually independent [5]. Since ligand screening has been typically performed by utilizing a G protein-mediated signal as the assay readout, monitoring the ß-arrestin-mediated pathway instead of G protein activation will increase the likelihood of identifying currently unknown ligands, including ß-arrestin-biased agonists.

In the present study, we developed a cell-based assay to monitor ligand-dependent GPCR-ß-arrestin interaction. An assay based on enzyme fragment complementation (EFC) is one of the best means to record this regulated protein—protein interaction [6–9]. In a reporter cell, complementary fragments of the enzyme (alpha and omega) are fused to ß-arrestin 2 and GPCR, respectively. Agonist stimulation initiates the interaction of these two fusion proteins (ß-arrestin 2-alpha and GPCR-omega), and this inducible interaction is monitored by the reconstituted enzyme activity. EFC assays have two important advantages. First, positive signals solely arise from the engineered targeted receptor containing one of the complementary enzyme fragments. This feature minimizes the assay backgrounds and makes the system specific to the targeted receptor activation. Second, the assay reaction of EFC produces a high-magnitude output signal through enzymatic amplification. EFC assays are therefore generally sensitive.

Since both of the complementary fragments are small (<19 kDa), ß-lactamase is an attractive reporter for EFC assays [8,10]. The ß-lactamase reporter is assayable with CCF4/AM, a highly sensitive cell-permeable fluorescent substrate, whereas other enzyme reporters such as ß-galactosidase, alkaline phosphatase and firefly luciferase, require cell permeabilization to load their fluorescent substrates into a reporter cell to perform fluorescence-activated cell sorting (FACS) [11]. As such, ß-lactamase provides a non-destructive assay, which is compatible with FACS. This enabled us to perform high-throughput screening for identifying individual live cell clones with defined responses. In terms of the compatibility with FACS, the green fluorescent protein (GFP) reporter is equally useful. However, the detection sensitivity of GFP is much lower due to the lack of enzymatic signal amplification. Thus, about 100 ß-lactamase molecules per cell are sufficient to detect a significant signal, whereas 10^5^ to 10^6^ GFP molecules per cell are required to exceed background autofluorescence [11]. Accordingly, we employed here a ß-lactamase EFC to monitor the agonist-induced GPCR-ß-arrestin interaction.

This assay system was applied to ligand screening of putative peptidergic orphan GPCRs. We screened peptide fractions extracted from various mammalian tissues, and discovered agonistic activities for human bombesin receptor subtype 3 (hBRS3) in bovine adrenal extracts. Peptide E, a partially processed product from the peptide precursor proenkephalin A, was subsequently purified as an endogenous agonist. These results demonstrate that our newly developed system is sufficiently robust for ligand screening of GPCRs.

## Materials and Methods

### Plasmids and cells

GPCR-omega chimera was subcloned into a pWZL-IRES-Neo retroviral vector and ß-arrestin 2-alpha chimera was subcloned into a pWZL-IRES-Puro vector. These plasmids were individually transfected into the retrovirus packaging cell line Plat E [12]. The supernatant from the transfected cells was removed 48–72 h later and applied to CHO cells expressing the ecotropic receptor. Transduced cells were selected and maintained in the presence of the appropriate antibiotics (neomycin 1mg/ml and puromycin 0.75μg/ml). CHO cells were grown in DMEM supplemented with 5% FCS and 1% nonessential amino acids (Invitrogen).

### ß-lactamase Assay

CHO cells stably expressing both the GPCR-omega chimera and ß-arrestin 2-alpha chimera were plated either in 6 well plates or in 96 well plates. After 24 h, ligands or HPLC fractions were added to the wells, and incubated for 14 h. After incubation, cells were washed once in HBSS and then incubated with CCF4/AM substrate for 2 h. ß-lactamase activity was measured either by FACS or with a plate reader (Victor V: Perkin Elmer).

### Purification of peptide E

Bovine adrenal glands (400 g, purchased from Pel-Freez Biologicals, AR 72756, USA) were diced and boiled in 10 volumes of water for 15 min to deactivate endogenous proteases. The solution was adjusted to 1M AcOH and homogenized. After 45 min centrifugation and filtration, the supernatant of the extracts was loaded onto OASIS HLB Extraction cartridges (Waters). The OASIS cartridges were washed with 10% CH_3_CN/0.1%TFA and eluted with 60% CH_3_CN/0.1%TFA. The eluate was lyophilized and redissolved in 20mM Na-phosphate (pH3.0)/30% CH_3_CN. Step1 (Fig 2A): the extract was applied to a cation-exchange HPLC column (TOSOH Bioscience SP-5PW; 21.5mm x 150mm), preequilibrated with 20mM Na-phosphate (pH3.0)/30% CH_3_CN at room temperature. A 0–0.5M gradient of NaCl in 20mM Na-phosphate (pH3.0)/30% CH_3_CN was applied over 60 min at a flow rate of 10 ml/min. Twenty-milliliter fractions were collected, and 5% of each fraction was set aside and assayed. Step 2: the active fractions were pooled, diluted with water and loaded onto a C18 reverse-phase HPLC column (Vydac 238TP510; 10mm x 250mm), preequilibrated with 20% CH_3_CN/0.1%TFA at a flow rate of 3 ml/min at room temperature. A 20%-35% gradient of CH_3_CN in 0.1%TFA was applied over 60 min. Fractions, each of 9 ml, were collected and 8% of each fraction was used for the assay. Step 3: the active fractions were pooled, diluted with water and applied to a C8 reverse-phase HPLC column (Waters Xterra RP8; 4.6mm x 250mm), preequilibrated with 20% CH_3_CN/0.1%TFA at a flow rate of 1 ml/min at room temperature. A 26%-33% gradient of CH_3_CN in 0.1%TFA was applied over 35 min. Fractions were collected and 10% of each fraction was used for the assay. Step 4 (Fig 2B): the active fractions were pooled, diluted with water and applied to a C18 reverse-phase HPLC column (Waters Symmetry Shield RP18; 4.6mm x 150mm), preequilibrated with 25% CH_3_CN/0.1%TFA at a flow rate of 1 ml/min at room temperature. A 25%-32% gradient of CH_3_CN in 0.1%TFA was applied over 35 min. The major peaks were collected manually and the structure of the purified material was determined by Edman microsequencing and tandem MS/MS analysis.

### Intracellular Calcium Transient Assay

CHO cells stably expressing GPCR of interest were loaded with Fura-2 AM in suspension. [Ca^2+^]i transients evoked by the ligands were monitored using a CAF-110 Intracellular Ion Analyser (JASCO) in 500 μl cuvettes as previously described [13].

### Luciferase Reporter Assay

CHO cells stably expressing the GPCR of interest were transiently transfected with NFAT-TA-Luc plasmid (Clontech). Cells were then left overnight before being plated in 96 well plates (20000 cells/well). After an additional 24 h, the synthetic ligands were added to the wells and incubated for 6 h. Following incubation, firefly luciferase assays were performed using the Steady-Glo Luciferase Assay System (Promega). Luminescence was detected with a plate reader (Victor V: Perkin Elmer), according to the manufacturer’s instructions. EC_50_ values were calculated using Workout 2.0 Data Analysis Software (Perkin Elmer).

### Radioligand Binding Assay

CHO cells expressing hBRS3 grown in 150 mm dishes were harvested in binding buffer (50mM Tris-HCl (pH7.5), 5mM MgCl_2_ and protease inhibitor cocktail) and disrupted with Dounce homogenization. After removal of cell nuclei and mitochondria by a low-speed centrifugation (8000 g x 15 min), the cell membrane fractions were collected by centrifugation at 50000 g for 60 min. The protein concentration was determined using a Bio-Rad protein assay kit (Bio-Rad). For the binding assay, 5 μg of membrane fraction was incubated with 0.1 nM of the radioligand ([D-Tyr[^125^I], ßAla^11^, Phe^13^, Nle^14^]- Bombesin (6–14); Perkin Elmer) and various concentrations of competitors [14]. After 2 h incubation at room temperature, the bound ligand was separated from the free ligand by filtering through a GF/C filter (Whatman) and washed twice with washing buffer (50mM Tris-HCl (pH7.5), 0.5mM EDTA and 0.2% BSA). The amount of the radioligand bound to the receptor was determined by measuring the radioactivity on the filter using a gamma-counter. The IC_50_ was calculated using Workout 2.0 Data Analysis Software (Perkin Elmer).

### PathHunter Assay

PathHunter assays for hBRS3 were performed as previously described [13].

### Statistical Analyses

Results are expressed as the mean ± SEM. Data involving multiple groups were assessed by ANOVA with Dunnett’s multiple comparison of means test.

## Results

### Establishing a reporter cell line that monitors the agonist-induced ß-arrestin-GPCR interaction via ß-lactamase EFC

**Fig 1:**
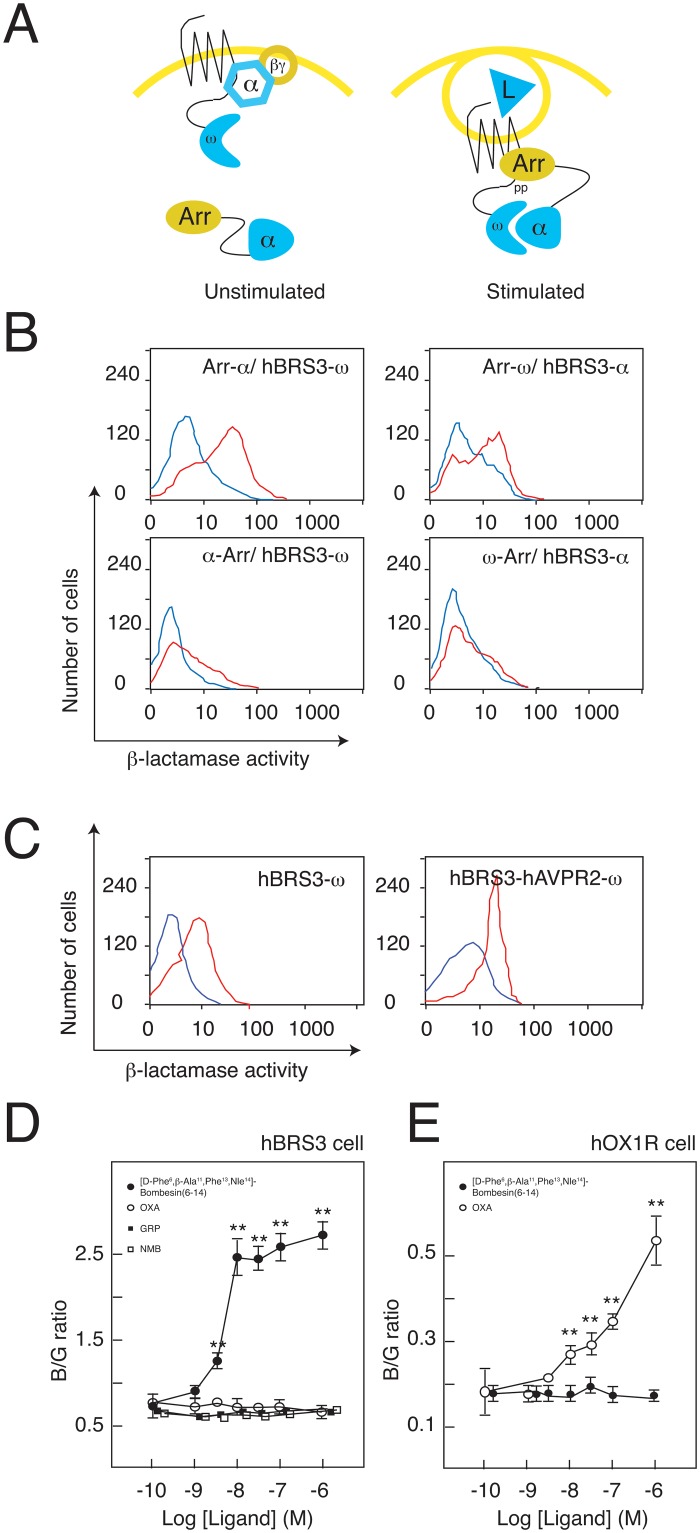
ß-lactamase EFC assay to monitor the agonist-induced GPCR-ß-arrestin interaction. (A) Schematic of the ß-lactamase EFC assay to monitor GPCR-ß-arrestin interaction. (B) FACS analyses of four different combinations of the fusion proteins. FACS histograms in the absence (blue line) and in the presence (red line) of ligand (10 nM) are overlaid. (C) FACS analysis of the hBRS3-hAVPR2 chimera. (D) Comparative dose-response of bombesin-related peptides to hBRS3 in the ß-lactamase EFC assay. (E) Comparative dose-response of orexin-A to OXIR in the ß-lactamase EFC assay. Representative data (mean ± SEM) from at least 3 independent experiments performed in triplicate are shown for (D) and (E). ** p<0.01, one-way ANOVA with Dunnett’s multiple comparison test.

Chimeric proteins were constructed with GPCRs, ß-arrestin 2 and the complementary ß-lactamase fragments (alpha and omega) (Fig 1A). A reporter cell line stably expressing two chimeric proteins was established through the sequential retroviral infection of CHO cells. We selected hBRS3 as a model receptor because it is an orphan receptor with a surrogate ligand ([D-Phe^6^, ß-Ala^11^, Phe^13^, Nle^14^]-Bombesin (6–14); Phoenix Pharmaceuticals, Inc.) [14,15]. In order to make the reporter cell line as sensitive as possible, we carried out three optimization steps:

First, we made all four combinations of chimeric proteins. The signal-to-noise (S/N) ratio of each combination was examined by flow cytometry. Among the four, hBRS3-omega with ß-arrestin-alpha was the best (Fig 1B).

Second, we examined if changing the C-terminal tail sequence of hBRS3 improved the sensitivity of the system, because the C-terminal tail of the receptor determines the binding affinity for ß-arrestins. Arginine vasopressin receptor 2 (AVPR2) is known to bind ß-arrestins with high affinity [16]. We thus made a chimeric hBRS3 whose C-terminal tail is swapped with that of hAVPR2. As expected, the hBRS3-hAVPR2 chimera showed a higher signal when stimulated with a surrogate ligand (Fig 1C). However, the baseline signal levels were also increased, resulting in no improvement of the S/N ratio (Fig 1C). Based on this result, we chose the original tail of hBRS3.

Third, we established and screened more than 50 monoclonal clones harboring two chimeric proteins (ß-arrestin-alpha, hBRS3-omega). Their responses to a surrogate ligand were individually tested and the clone with the best S/N ratio (clone #6–3) was isolated.

These optimization steps enabled us to make the system as sensitive as other functional assays. This cell line (#6–3) responded to a surrogate ligand specifically at nanomolar concentrations, but not to other mammalian bombesin-like peptides such as gastrin-releasing peptide (GRP) and neuromedin B (NMB), nor unrelated peptides such as orexin-A (Fig 1D). To evaluate the possibility that the signals produced by the surrogate ligands (Fig 1D) were skewed due to the effects of fusioning ß-lactamase fragments to receptor or ß-arrestin, we also performed PathHunter ß-arrestin assays. The PathHunter assay is well characterized and a widely used system in this field. Surrogate ligands also recruited ß-arrestins to hBRS3 in the PathHunter assay, and a similar dose-response curve was obtained (S1 Fig).

We also generated a reporter cell line for orexin type 1 receptor (OX1R), and used it as a negative control for ligand screening. OX1R cells responded specifically to orexin-A as expected (Fig 1E). Before initiating screening, we challenged the OX1R reporter cells with high-performance liquid chromatography (HPLC) fractions extracted from bovine hypothalamus, where the orexin peptides are abundantly produced [17]. We detected significant activity in the fraction where orexin peptide would be eluted, but no activity was observed in other fractions (S2 Fig). This preliminary experiment thus validated the sensitivity and specificity of our assay system.

### Purification of peptide E as an endogenous ligand for hBRS3

**Fig 2:**
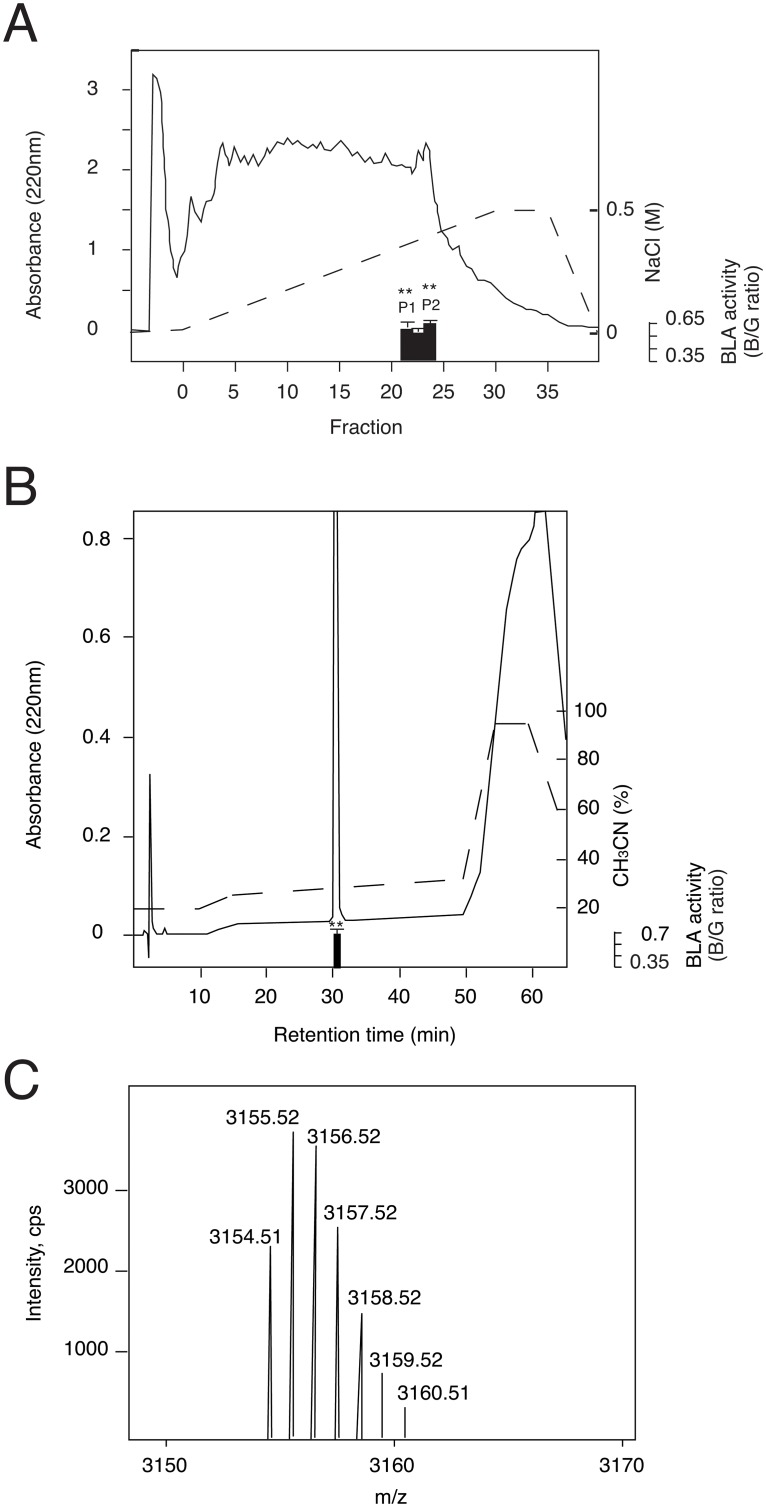
Purification of peptide E from bovine adrenal glands. (A and B) HPLC profiles of the first step using a cation-exchange column (TOSOH Bioscience SP-5PW; 21.5mm x 150mm) (A), and of the last step using a C18 reverse-phase column (Waters Symmetry Shield RP18; 4.6mm x 150mm) (B) with the ß-lactamase assay results. (C) (M+H)^+^ ions in the electrospray ionization mass spectrum of the purified peptide. Representative data (mean ± SEM) from at least 3 independent experiments performed in triplicate are shown for (A) and (B). ** p<0.01, one-way ANOVA with Dunnett’s multiple comparison test.

**Fig 3:**
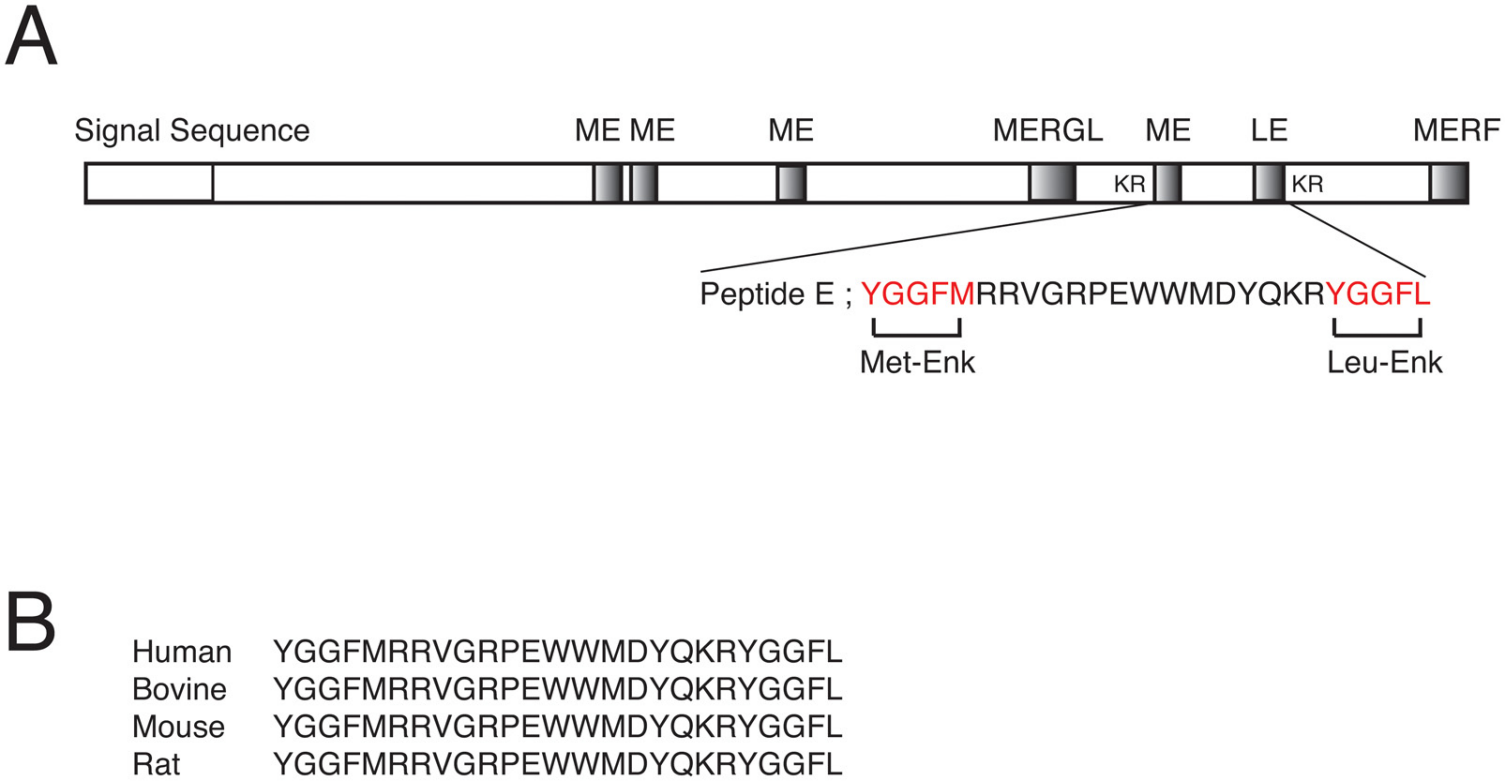
The amino acid sequence of peptide E. (A) The structure of the peptide precursor proenkephalin A, and the location of peptide E in the precursor. (B) Peptide E sequences from various species are aligned.

Using #6–3 cells, we screened peptide fractions extracted from various mammalian tissues. Two specific and significant activities were found in cation-exchange HPLC fractions from bovine adrenal extracts (Fig 2A). We isolated two active substances to homogeneity after the three successive steps of reverse phase HPLC as described under *Materials and Methods* (Fig 2B). The final materials were subjected to structural analyses by Edman microsequencing and tandem MS/MS. Monoisotopic mass of the active substance corresponding to P2 was 3154.5 Da (Fig 2C). Combined with Edman microsequencing and tandem MS/MS results, the peptide sequence of P2 was determined to be YGGFMRRVGRPEWWMDYQKRYGGFL. The substance corresponding to P1 was the methionine sulfoxide form of P2. This amino acid sequence exactly matches that of peptide E, which is encoded in the *proenkephalin A* gene [18]. Peptide E is an intermediate opioid peptide produced from the opioid peptide precursor proenkephalin A by partial processing (Fig 3A) [19]. It has 25 amino acid residues with a Met-enkephalin sequence at the N-terminus and a Leu-enkephalin sequence at the C-terminus. In the proenkephalin A precursor, both ends of peptide E are flanked with a pair of basic amino acid residues, which are typically recognized and cleaved by the pro-hormone convertases. Interestingly, the amino acid sequence of peptide E is well conserved among different species, suggesting that peptide E may function not only as an intermediate for mature opioid peptides, but also as a bioactive signaling molecule *per se* (Fig 3B).

### In vitro pharmacology of peptide E on the mammalian bombesin receptors

**Fig 4:**
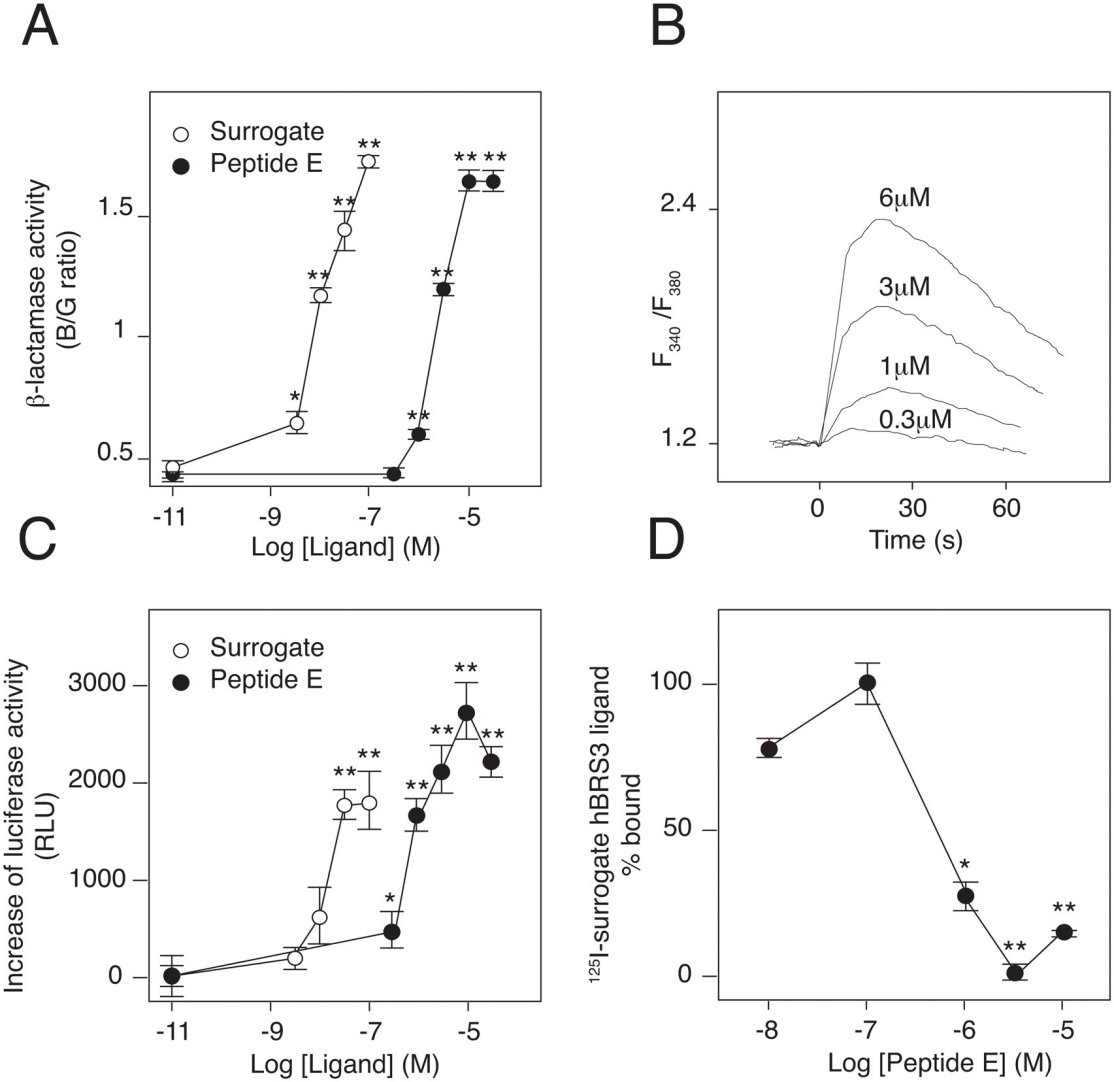
*In vitro* pharmacology of peptide E on hBRS3. (A) Dose-response of peptide E to hBRS3 in the ß-lactamase EFC assay. (B) Peptide E stimulates [Ca^2+^] transients in CHO cells expressing hBRS3. (C) Dose-dependent activation of NFAT-promotor by peptide E in CHO cells expressing hBRS3. Peptide E and the surrogate ligand activated hBRS3 with EC_50_ values of 500 nM and 8.8 nM, respectively. (D) Competition of the ^125^I-synthetic hBRS3 surrogate ligand binding to the membrane fractions of CHO-hBRS3 cells by peptide E. The IC_50_ was calculated to be 361 nM. Representative data (mean ± SEM) from at least 3 independent experiments performed in triplicate are shown. * p<0.05, ** p<0.01, one-way ANOVA with Dunnett’s multiple comparison test.

To confirm its pharmacological activities, peptide E was chemically synthesized. As expected, synthetic peptide E increased ß-lactamase activity in #6–3 cells in a dose-dependent manner (Fig 4A and S3 Fig). Using CHO cells stably expressing hBRS3, the agonistic activity of peptide E was further confirmed in [Ca^2+^]i transient assays (Fig 4B and S4 Fig) and luciferase reporter assays driven by the nuclear factor of activated T cells (NFAT) promoter (Fig 4C and S4 Fig). The half maximal effective concentration (EC_50_) was determined to be 500 nM in the NFAT-luciferase assay. To demonstrate the direct interaction of peptide E with hBRS3, the competitive binding assay was also performed (Fig 4D).

**Fig 5:**
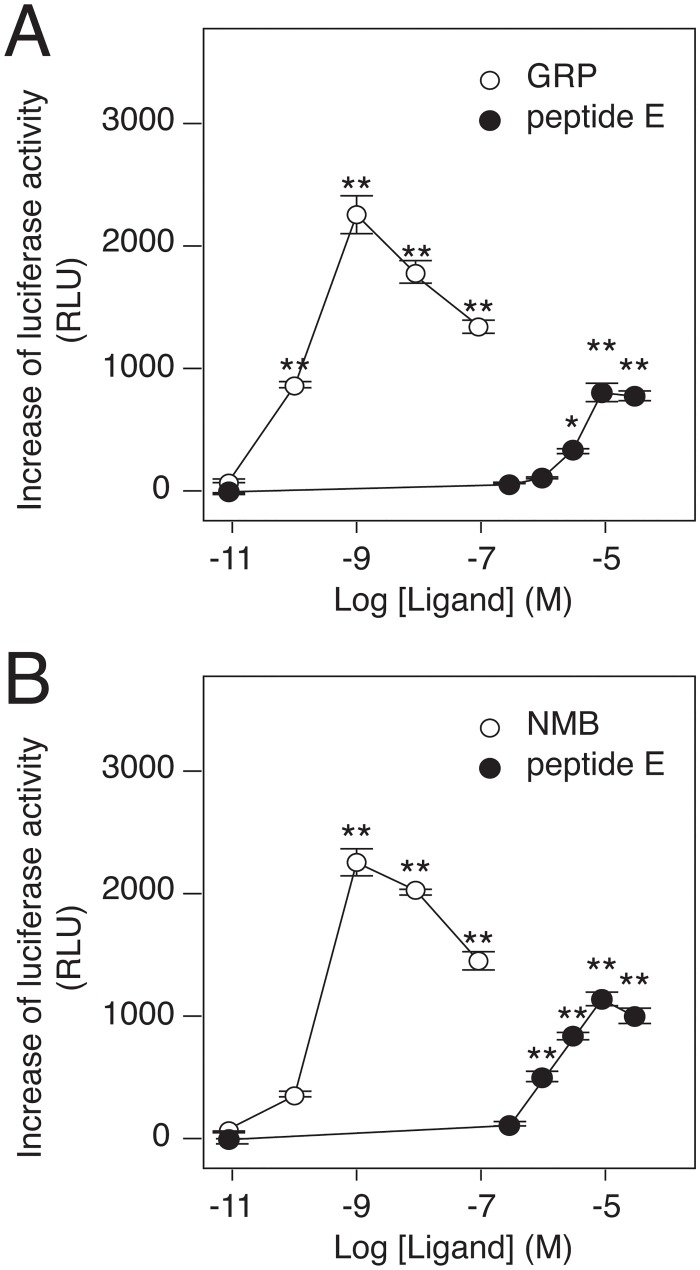
Agonistic activities of peptide E on hGRPR and hNMBR. (A) Peptide E activates NFAT-promotor in a dose-dependent manner in CHO cells expressing hGRPR. The EC_50_ values for peptide E and GRP were calculated to be 3.5 μM and 0.12 nM, respectively. (B) Peptide E activates NFAT-promotor in a dose-dependent manner in CHO cells expressing hNMBR. The EC_50_ values for peptide E and NMB were calculated to be 1.1 μM and 0.16 nM, respectively. Representative data (mean ± SEM) from at least 3 independent experiments performed in triplicate are shown. * p<0.05, ** p<0.01, one-way ANOVA with Dunnett’s multiple comparison test.

Next, we tested the pharmacological activities of peptide E on the other two mammalian bombesin receptors, gastrin-releasing peptide receptor (GRPR) and neuromedin B receptor (NMBR). Agonistic activities of peptide E on both receptors were revealed in NFAT-luciferase assays, using CHO cells overexpressing GRPR and NMBR, respectively (Fig 5). Although the potency of peptide E was weaker than that of their authentic high-affinity ligands, these results suggest that peptide E is an endogenous ligand for the mammalian bombesin receptors.

### Structure-activity relationship study

**Fig 6:**
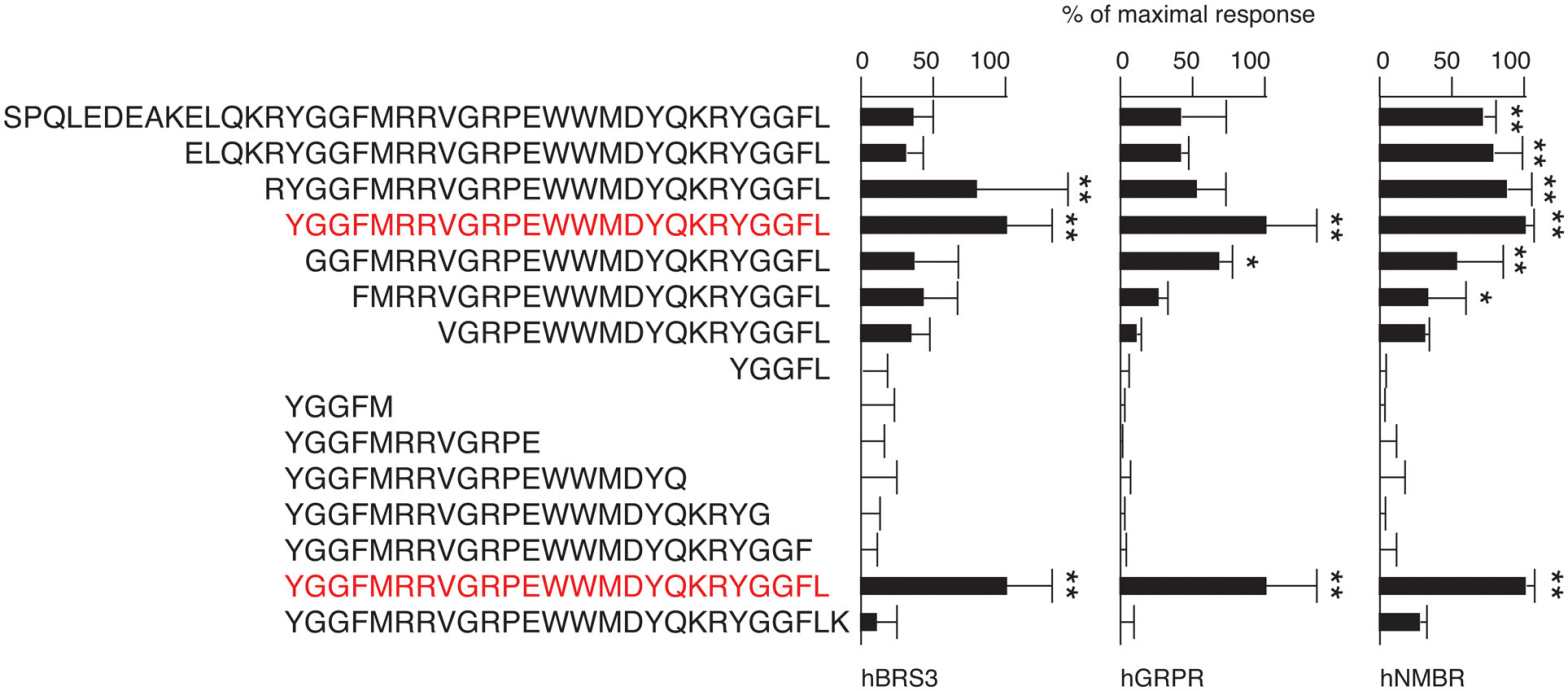
Structure-activity relationship study. The activities of various peptide E mutants (3 μM) on the mammalian bombesin receptors (hBRS3, hGRPR and hNMBR) were tested in NFAT-luciferase assays. Representative data (mean ± SEM) from at least 3 independent experiments performed in triplicate are shown. * p<0.05, ** p<0.01, one-way ANOVA with Dunnett’s multiple comparison test.

We studied the structure-activity relationship of peptide E on the mammalian bombesin receptors. Various peptide E mutants (N-terminally deleted and C-terminally deleted mutants) listed in Fig 6 were tested. Among them, peptide E was found to have the strongest activity.

Peptide E is an intermediate opioid peptide that activates all three opioid receptors (delta, kappa and mu opioid receptors) with nanomolar affinity, and its N-terminal enkephalin sequence (YGGFM) is known to be required for its opioid activity [13]. Interestingly, however, the N-terminal enkephalin motif is dispensable for the activation of the mammalian bombesin receptors, while the C-terminal structure of peptide E is critical (Fig 6). These results support the notion that opioid receptors and bombesin receptors, which constitute distinct GPCR subfamilies, have a different structural requirement for their activation.

## Discussion

Upon ligand binding, GPCRs recruit ß-arrestins, and this reversible interaction is independent of interacting G proteins. Based on this concept, we developed a cell-based assay that enabled us to monitor the agonist-induced GPCR-ß-arrestin interaction through ß-lactamase EFC.

In EFC assays, positive signals arise exclusively from the two engineered proteins that are fused to each of the two complementary enzyme fragments. As such, our newly developed assay is immune to intracellular signaling produced by the endogenous molecules expressed in a reporter cell. This feature minimizes the assay backgrounds and makes the system advantageous, particularly when crude samples, such as tissue extracts, are screened or if the ligand is a low-affinity and/or a partial agonist that weakly activates the receptor. Indeed, the minimized background of this system enabled us to purify peptide E as a low-affinity agonist for the mammalian bombesin receptors from bovine adrenal extracts. As far as we know, this is the first demonstration that a ß-arrestin recruitment assay was utilized for the biochemical purification of an endogenous GPCR ligand from the crude tissue extracts. Since successful purification of peptide E has validated the robustness of this assay, applying our methodology to other orphan GPCRs, or even to already de-orphanized receptors, should lead to the discovery of novel endogenous ligands that have been missed by the conventional assays for G protein signaling.

Recent studies have demonstrated that G protein- and ß-arrestin-mediated pathways are mutually independent [5]. Consistent with this, some GPCR ligands (G protein-biased and ß-arrestin-biased agonists) differentially activate either G protein- or ß-arrestin-mediated signaling. Since ß-arrestin signaling has physiological functions that are distinct from those of G protein signaling, the biased agonism of GPCRs has significant therapeutic implications [20]. For example, two different chemicals acting on the same GPCR might have distinct pharmacological effects *in vivo* due to varying degrees of bias toward the G protein- and ß-arrestin-mediated pathways [21]. This indicates that identification of GPCR biased ligands, which have been already validated as therapeutic targets, is a promising strategy for drug development. The system that we describe here will aid in the discovery of ß-arrestin-biased agonists, which have the potential to become new drugs.

It is well established that bioactive peptides, such as peptide hormones and neuropeptides, are synthesized as high molecular weight precursors and subsequently cleaved into bioactive forms by post-translational processing [22]. Precursor processing serves as a mechanism for amplification. Proenkephalin A is an example: several different opioid peptides are proteolytically cleaved from the one large precursor proenkephalin A.

Precursor processing also increases the diversity in bioactive peptide production. It is known that unrelated peptides with distinct functions are sometimes encoded in the same precursor. Proopiomelanocortin (POMC) is an example of such a precursor, from which alpha-MSH and adrenocorticotropic hormone (ACTH), the peptide ligands for melanocortin receptors, in addition to the opioid peptide ß-endorphin, are produced from the same precursor by differential processing. In this study, we unexpectedly determined that peptide E, one of the partially processed products from the opioid peptide precursor proenkephalin A, functions as a low-affinity agonist for the mammalian bombesin receptors. Since proenkephalin A is known to produce opioid peptides including enkephalins, the biological effects of proenkephalin A are generally considered to be mediated by the classical opioid receptors (i.e., mu, delta and kappa). However, some of these effects are resistant to naloxone, an opioid receptor antagonist, suggesting the involvement of receptors other than the opioid receptors [23]. We now speculate that some of these non-opioid functions of proenkephalin A may be mediated by the mammalian bombesin receptors. Although peptide E is a low-affinity ligand, our finding is another example showing that peptides, which act on distinct receptors (the opioid receptors and the bombesin receptors in this case), can be produced from the same peptide precursor (proenkephalin A) by differential processing.

As peptide E activates the mammalian bombesin receptors with low affinity, there are two possibilities for the functions of peptide E under physiological conditions. First, peptide E could act as an autocrine/paracrine factor. Proenkephalin A is one of the most abundantly expressed prohormone genes in the central nervous system [24]. Accordingly, if the volume of distribution of peptide E was very limited, local concentrations of this peptide could become sufficiently high to activate the mammalian bombesin receptors. Second, the bombesin receptors may form a protein-complex with putative accessory molecules to create a high-affinity binding site for peptide E. This hypothesis is supported by the existence of a family of single transmembrane proteins called receptor activity modifying proteins (RAMPs) [25]. Calcitonin receptor-like receptor (CRLR) by itself is known to bind to calcitonin gene-related peptide (CGRP) and adrenomedullin with low affinity. Interestingly, however, CRLR interacts with RAMPs, and this heterodimeric membrane-protein complex provides a high-affinity binding site for CGRP and adrenomedullin. Evaluation of these two intriguing possibilities may thus help determine the physiological roles for this newly identified ligand-receptor pair.

In summary, we have established a cell-based assay system for monitoring ß-arrestin recruitment via ß-lactamase EFC. Successful purification of peptide E as an endogenous ligand for the mammalian bombesin receptors has validated the robustness of this assay. We conclude that this newly developed system will thus facilitate the discovery of other GPCR ligands.

## Supporting Information

S1 Fig Dose-response effects of the surrogate ligand and peptide E on hBRS3 in the PathHunter assay. 293T cells stably expressing ß-arrestin 2-omega (omega: M15 deletion mutant of ß-galactosidase) were transiently transfected with hBRS3-alpha (a short alpha peptide fragment of ß-galactosidase). Cells were stimulated with various concentrations of the surrogate ligand and peptide E. Gal-Screen substrate was then added to the wells, and luminescence was measured in a plate reader (Victor V: Perkin Elmer). Representative data (mean ± SEM) from at least 3 independent experiments performed in triplicate are shown. ** p<0.01, one-way ANOVA with Dunnett’s multiple comparison test.
(EPS)

S2 Fig Screening of bovine hypothalamic fractions for agonistic activities on hOX1R using the ß-lactamase EFC assay. Bovine hypothalamic peptide fractions were extracted and separated under the same protocol for the preparation of bovine adrenal fractions. These fractions were then tested using hOX1R cells for the ß-arrestin recruitment assay. Representative data (mean ± SEM) from at least 3 independent experiments performed in triplicate are shown. ** p<0.01, one-way ANOVA with Dunnett’s multiple comparison test.
(EPS)

S3 Fig Peptide E shows no activity against hOX1R in the ß-lactamase EFC assay. hOX1R cells for monitoring ß-arrestin recruitment were stimulated with various concentrations of peptide E under the protocol described in the Materials and Methods section.
(EPS)

S4 Fig Untransfected wild type CHO (WT-CHO) cells do not respond to peptide E. WT-CHO cells and WT-CHO cells harboring NFAT-luciferase reporter were stimulated with peptide E. No calcium transient or luciferase activity was induced by peptide E stimulation.
(EPS)
